# To Our Patrons

**Published:** 1859-11

**Authors:** 


### EDITORIAL AND MISCELLANEOUS.

### To Our Patrons.

The present number of the Journal was printed and ready for distribution at the time of the fire, which consumed the office in which it was published. The principal part of the printed sheets were also consumed, and we have been under the necessity of re-printing the number, which will account for the delay in sending it out to our subscribers. The December number will be issued at as early a day as possible.

